# Psychometric evaluation of the anticipatory grief scale in a sample of family caregivers in the context of palliative care

**DOI:** 10.1186/s12955-019-1110-4

**Published:** 2019-03-05

**Authors:** Maja Holm, Anette Alvariza, Carl-Johan Fürst, Joakim Öhlen, Kristofer Årestedt

**Affiliations:** 1 Department of Nursing Sciences, Sophiahemmet University, Box 5605, 114 86 Stockholm, Sweden; 2 Department of Health Care Sciences, Ersta Sköndal University College, Box 11189, 100 61 Stockholm, Sweden; 3 Capio Geriatrics, Palliative care unit, Dalen hospital, Åstorpsringen 6, 121 87, Stockholm, Sweden; 4 Department of Clinical Science and the Institute for Palliative Care, Lund University, Scheelevägen 2, 223 81 Lund, Sweden; 5 Institute of Health and Care Sciences, Sahlgrenska Academy, University of Gothenburg, Box 457, 405 30 Gothenburg, Sweden; 6 Centre for Person-Centred Care, University of Gothenburg, Arvid Wallgrens backe 1, 413 46 Gothenburg, Sweden; 7 Faculty of Health and Life Sciences, Linnaeus University, 391 82 Kalmar, Sweden; 8 The Reserch Section, Region Kalmar County, Lasarettsvägen 1, 392 44 Kalmar, Sweden

**Keywords:** Anticipatory grief, Palliative care, Family caregivers, Instrument development, Factor analysis, Nursing

## Abstract

**Introduction:**

In palliative care, family caregivers are often faced with experiences of grief in anticipation of the loss of a close person. An instrument designed to measure this form of grief is the Anticipatory Grief Scale, which includes 27 items and has been used in several studies in various contexts. However, the instrument has not been validated.

**Aim:**

The aim was to evaluate the psychometric properties, focusing on the factor structure, of the Anticipatory Grief Scale in a sample of family caregivers in palliative care.

**Methods:**

The study had a cross-sectional design. Data were collected from an intervention study in palliative home care that took place between 2013 and 2014. In total, 270 family caregivers in palliative care completed a baseline questionnaire, including the Anticipatory Grief Scale. The factor structure of the scale was evaluated using exploratory factor analysis.

**Results:**

The initial factor analysis suggested a four-factor solution, but, due to weak communalities, extensive crossloadings, and item inconsistencies, the model was problematic. Further analysis supported that the scale should be reduced to 13 items and two factors. The two subscales captured the behavioral and emotional reactions of grief in family caregivers in palliative care and were named *Behavioral reactions* and *Emotional reactions.* This modified version will hereafter be named AGS-13.

**Conclusions:**

This validation study of the Anticipatory Grief Scale resulted in a revised two-factor model, AGS-13, that appears to be promising for use in palliative care but needs to be tested further.

## Background

Grief is generally defined as the psychological and physiological response to the death of a close person [1]. Grief is, in itself, not pathological, but the reactions and consequences of it can be [2]. Grief before a close person’s death has been conceptualized as anticipatory grief, a term that was first defined by Lindemann in light of the Freudian psychoanalytic theory. Anticipatory grief was seen as a form of ‘grief work’ before an actual loss, where the grieving person would gradually detach their bonds to the dying person [3]. This understanding of the concept of anticipatory grief has since been expanded, particularly in relation to palliative care, where family caregivers may face a complex and stressful situation. They often spend a considerable amount of time caring for the patient [4] and their efforts are often indispensable to the health care system [5]. In order to promote efficient support, it has been stated that health care professionals should be attentive to anticipatory grief reactions in family caregivers [6] due to its potential consequences, such as emotional stress, loneliness, cognitive dysfunction, and social withdrawal [7]. Hence, there is a need to find methods to identify and measure anticipatory grief in family caregivers of patients who are in receipt of palliative care.

Of late, the concept of anticipatory grief has been discussed and sometimes renamed ‘pre-death grief’ or ‘pre-loss grief’ because it merely indicates the presence of grief symptoms before a person’s death rather than anticipation of bereavement [8]. Other studies have indicated that there are some differences between these concepts, although they are often used interchangeably [9]. A recent review defined anticipatory grief as a multidimensional concept and a dynamic process [10]. Nuclear constructs of anticipatory grief have been described as anger, guilt, anxiety, irritability, sadness, feelings of loss, and decreased ability to function in performing usual tasks [11]. According to the original theory, anticipatory grief would improve bereavement outcomes because the grief work had already been commenced before the loss [3]. However, in later years, the theory of grief work has been questioned [12], and recent results indicate that experiencing high levels of anticipatory grief is associated with low preparedness for the loss, and additional problems in bereavement, such as complicated grief and post-loss depression [13, 14].

There are several different instruments constructed to measure anticipatory grief. Some of them are designed as ‘pre-death’ versions of instruments that are otherwise created to measure post-bereavement grief and these differentiate between normal or pathological grief. Examples are the pre-loss version of the PG-13 and the Pre-death Inventory of Complicated Grief [15, 16]. A recent review found six different instruments in 14 studies to measure anticipatory grief, and no more than three studies used the same instrument. Although they all aimed to measure caregivers’ emotional status, there were substantial differences between the instruments. The authors of the review concluded that there is a lack of common understanding of the concept of anticipatory grief and a dearth of psychometric testing of the instruments used to measure the phenomenon [8].

One of the more widely used instruments designed to measure anticipatory grief is the Anticipatory Grief Scale (AGS), which was developed by Theut, Jordan, Ross, and Deutsch (1991). The AGS represents the major domains cited in the literature on grief. The items are constructed based on a combination of clinical experience and other instruments measuring grief, such as the Texas Revised Inventory of Grief (TRIG), which has been developed to measure grief in bereavement [17]. According to Theut et al., the AGS also investigates the reactions of grief, but before the loss. The AGS was originally developed as to be used on family caregivers of patients affected by dementia, however, the wording could be changed and used in other contexts, such as in cancer and palliative care [7, 18–20]. Although it has been used internationally, one important limitation is that the AGS has not been rigorously validated. Most important, even if AGS is constructed as a unidimensional measure of anticipatory grief, no previous study has evaluated the factor structure of the instrument, which is an important aspect of construct validity. Moreover, no previous study has evaluated the AGS conceptually for example by correltating the scores with similar and closely related concepts such as grief, anxiety or depression. For further use in palliative care, the AGS also needs to be psychometrically evaluated when used with family caregivers of patients in palliative care. Hence, the aim of this study was to evaluate the psychometric properties, focusing on factor structure, of the Anticipatory Grief Scale in a sample of family caregivers in palliative care.

## Methods

### Design

This psychometric evaluation study was based on the baseline data from a previously conducted intervention trial, one that aimed to increase family caregivers’ preparedness for providing palliative care. Details about the intervention study are presented elsewhere.

### Participants and procedure

The study was conducted in the context of specialized palliative home care in a metropolitan area in Sweden. In all, 10 palliative care settings were involved. The palliative care provided by the home care settings included advanced symptom relief, palliative treatments and existential and emotional support. The settings were organized in multi-professional teams, including nurses, physicians, social workers and occupational and physical therapists. The inclusion criteria for the study were: being a family caregiver to a person in palliative care, over the age of 18, and being able to understand the Swedish language. Designated health care professionals at each setting were responsible for the recruitment of family caregivers over a period of fifteen months in 2013 and 2014.

Patients were initially approached by the health care professionals and asked to agree that their family caregiver would be invited to participate in the study. They were also asked to consent to some information being taken from their medical records about their diagnosis and time of illness. If the patient agreed, the family caregiver was invited to participate in the study and was asked to complete a questionnaire, which included the AGS and additional questions about demographic background data. The questionnaire was returned to the research team by post.

### Instruments

The questionnaire consisted of demographic questions (sex, age, social and economic situation and educational level) and self-rating scales, among them a Swedish version of the AGS. The questionnaire also included the Hospital Anxiety and Depression scale (HADS). In a later stage, after the patient’s death, family caregivers answered the Texas Revised Inventory of Grief (TRIG). The two later instruments were used for construct validity purposes in the present study.

The AGS-scale consists of 27 items measuring anticipatory grief on a Likert scale, ranging from 1 (strongly disagree) to 5 (strongly agree). According to the constructor, the AGS items should be summed into a total score ranging from 27 to 135 with a higher score indicating higher levels of anticipatory grief. Eight items (2, 5, 8, 11, 19, 22, 26, 27) have a positive bearing and therefore must be reversed before the total score is calculated. No subscales or cut-off scores have been reported for the AGS. The original English version of the AGS has previously been translated into Swedish by a research group led by Associate Professor Grimby at Sahlgrenska Academy (unpublished). The group employed a standard procedure for translation and back-translation using two independent bilingual language experts. For the present study, several attempts have been made to communicate the validation process with the original authors, however, these attempts have been unsuccessful.

The Hospital Anxiety and Depression scale (HADS) has been developed to measure anxiety and depression over two subscales. The instrument consists of 14 items, of which seven measure anxiety and depression respectively. The questions are answered on a four point response scale ranging between 0 and 3. The total score for each scale range between 0 and 21 where higher scores indicate more problems with anxiety or depression. The scale has shown satisfactory psychometric properties in different samples and language versions including the Swedish version [21].

The Texas Revised Inventory of Grief (TRIG) has been developed to measure the intensity of grief after bereavement. The instrument consists of two scales. TRIG I has eight items and involves thinking back to the time immediately after the loss (past behavior). TRIG II has 13 items and involves the bereaved person’s current grief reactions (present feelings). The items are measured on a Likert-type scale between strongly agree (1) and strongly disagree (5). The total score ranges between 8 and 40 and 13–65 for TRIG I and TRIG II respectively. Lower scores indicate more intense grief reactions. The scale has shown satisfactory psychometric properties in family caregivers to patients in palliative care [22].

### Analysis

Descriptive statistics were used to present demographic data and study variables.

All statistical analyses of the AGS was made after the scores of items with positive bearing had been reversed. Data quality was evaluated regarding the distribution of item and scale scores, and missing data patterns. Floor and ceiling effects for items, which refer to the proportions of participants with the lowest (floor) and highest (ceiling) possible scores, were evaluated using frequency distributions. A floor/ceiling effect of up to 20% was considered acceptable. The D’Agostino test, including skewness and kurtosis statistics, was conducted to evaluate whether the scale scores deviated significantly from the normal distribution. A normal distribution has skewness and kurtosis values close to 0 and 3, respectively, and a *p*-value ≥0.05. A graphic examination of the scale score distribution was also conducted using normal probability plots (P-P and Q-Q plots). Missing data patterns were evaluated using percentages. Having up to 5% missing data was considered acceptable. Spearman’s rank order correlation (*r*_*s*_) was used to evaluate associations between scale scores.

Homogeneity was evaluated with inter-item correlations and item-total correlations, using polychoric and polyserial correlations respectively (rho). Inter-item correlations between 0.15–0.85 and item-total correlations > 0.3 support homogeneity [23]. As calculations of polychoric correlations rely on an assumption of bivariate normality for the latent variables measured on a ordinal scale, a specific RMSEA test, developed and described by Jöreskog was applied to verify that there was no violation of this assumption. The RMSEA test for all pairs ranged between 0.000 and 0.091 which is below the recommended level of < 0.10 [24]. Therfore, the polychoric correlations are probably unbiased and can be deemed as reliable coefficients.

The factor structure of the AGS was examined through exploratory factor analyses. The Kaiser-Meyer-Olkin test (KMO = 0.84) and Bartlett’s test (χ^2^(351) = 2270.3, *p* < 0.001) supported the hypothesis that the data were suited for a factor analysis. As the items were treated as ordinal data, an unweighted least square (ULS) estimation method was used, and a polychoric correlation matrix was analyzed. A hot-deck multiple imputation was conducted for 14 participants who had incomplete data in the AGS. Using the same correlation matrix, a parallel analysis, based on optimal implementation and 500 replications, was conducted to identify the number of relevant factors to extract. To facilitate the interpretation of the factors, an ortogonal (varimax) rotaion was applied [25]. In the first step, a model that included all 27 items was conducted. A revised model was examined in a second step. Factor loadings, communality values (*h*^2^), the residual correlation matrix, and the Goodness of Fit Index (GFI) were all used to evaluate the models. To support model fit, factor loadings should be > 0.3 on the actual factor, communality values should be > 0.3, and GFI should be > 0.95.

The internal consistency was evaluated using an ordinal variant of Cronbach’s alpha, which is based on polychoric correlation rather than Pearson’ correlations [26]. The interpretation should be regarded equally, i.e., alpha should be > 0.7 [27]. For comparisons with previous studies, traditional alpha values were calculated.

Construct validity was evaluated through Spearman’s rank correlations (*r*_s_). Previous studies have shown that grief before death is associated with anxiety and depressive symptoms as well as grief after death. It was therefore hypothesized that AGS should correlate moderately to strongly with the HADS- anxiety, HADS depression and TRIG I and II (*r*_s_ = 0.4–0.8). Because lower scores on TRIG indicates more grief, unlike AGS where lower scores indicates less grief, we expected negative correlations between these scales.

The FACTOR 10.3 (Rovira Virgili University, Tarragona, Spain) was used to perform the the factor analysis while LISREL 8.80 (Scientific Software International, Inc., Skokie, IL, USA) was used to test the assumptions of bivariate normality for polychoric correlations. All other analyses were conducted using the R 3.5.1 (The R Foundation for Statistical Computing, Vienna, Austria), including the PSYCH package. The level of statistical significance was overall set at *p* < 0.05.

## Results

### Characteristics of family caregivers

In total, 270 family caregivers agreed to complete the AGS questionnaire. Most of them were women (68%) and they had a mean age of 61.0 (SD = 14.0) years. A majority (75%) were family caregivers to a patient affected by cancer (Table 1).

**Table 1:** Characteristics of participants (*n* = 270)

Age, *m* (SD)	61.0 (14.0)
Gender, *n* (%)	
Men	86 (31.8)
Women	184 (68.2)
Social status, *n* (%)	
Married/in partnership	192 (71.1)
Unmarried	78 (28.9)
Education, *n* (%)	
University	118 (43.7)
Non-university	152 (56.3)
Employment, *n* (%)	
Employed	134 (49.6)
Retired	109 (40.4)
Other	27 (10.0)
Living with patient, *n* (%)	
Yes	143 (53.0)
No	127 (47.0)
Relation to patient, *n* (%)	
Spouse	137 (50.7)
Adult child	94 (34.8)
Other	39 (14.5)
Patient diagnosis, *n* (%)	
Cancer	202 (74.8)
Other	68 (25.2)

### Item and scale score statistics

Half of the items of the AGS either had a pronounced negative skew (4, 6, 12, 15, 17, 22) or a positive skew (2,7, 9, 13, 18, 20, 21, 24) distribution, reflected by the median values and score distribution. Floor effects were demonstrated for 12 items and ceiling effects for 8 items. Problems with missing data were few between 0.3 and 1.5% across items (Table 2).

**Table 2:** Item statistics for the Anticipatory Grief Scale (n = 270)

	Score distribution, % ^a^							
	Strongly disagree (1)	Disagree (2)	Somewhat agree (3)	Agree (4)	Strongly agree (5)	Missing data	Mdn (q1-q3)	ITC ^b^
1. I daydream about how life with my relative was before the diagnosis of incurable illness was made.	15.2	14.8	29.0	20.4	**20.4**	0.4	3 (2–4)	0.603
2. I feel close to my relative who has incurable illness.	0.74	0.0	7.44	27.8	**63.7**	0.8	5 (4–5)	0.069
3. I seem to be more irritable since the diagnosis of incurable illness was made for my relative.	**20.7**	24.4	28.5	17.8	8.2	0.3	3 (2–4)	0.565
4. I am preoccupied with thoughts about my relative and his or her illness.	1.9	9.6	23.7	30.4	**33.7**	0.7	4 (3–5)	0.565
5. I have discovered new personal resources since my relative’s illness was diagnosed.	11.1	21.1	27.8	25.9	13.7	0.3	3 (2–4)	−0.094
6. I very much miss my relative the way he or she used to be.	5.6	11.1	19.6	23.0	**40.4**	0.4	4 (3–5)	0.442
7. I have felt very much alone since the diagnosis of incurable illness was made for my relative.	**27.8**	28.2	22.2	11.5	9.6	0.7	2 (1–3)	0.626
8. I am able to move ahead with my life.	3.7	10.7	29.6	35.9	18.5	1.5	4 (3–4)	0.453
9. I blame myself for my relative’s illness.	**81.1**	11.1	4.4	1.9	1.1	0.8	1 (1–1)	0.444
10. I find it hard to concentrate on my work since my relative was diagnosed with incurable illness.	**20.7**	20.4	33.0	19.0	6.7	0.4	3 (2–4)	0.668
11. I have the personal resources to help me cope with my relative and his or her illness.	2.6	10.8	35.2	38.6	11.9	1.1	4 (3–4)	0.444
12. I have periods of tearfulness as I think about my relative’s illness.	4.4	8.5	18.5	25.9	**42.2**	0.3	4 (3–5)	0.583
13. I feel detached from my relative.	**62.6**	24.4	9.6	2.2	0.3	0.7	1 (1–2)	0.456
14. I feel a need to talk to others regarding my relative’s illness.	10.0	11.1	33.7	30.0	14.8	0.4	3 (3–4)	0.315
15. I feel it is unfair that my relative has incurable illness.	10.4	11.9	13.7	20.4	**43.0**	0.7	4 (3–5)	0.449
16. I find it hard to sleep since my relative was diagnosed with incurable illness.	19.0	21.1	34.1	14.9	10.8	0.8	3 (2–4)	0.637
17.No one will ever take the place of my relative in my life.	5.2	5.6	16.7	15.2	**57.0**	0.4	5 (3–5)	0.208
18. I avoid some people since my relative was diagnosed with incurable illness.	**62.6**	15.6	10.4	8.2	3.0	0.4	1 (1–2)	0.552
19. I feel I have adjusted to my relative’s illness.	3.3	8.2	35.2	32.6	**20.4**	0.4	4 (3–4)	0.145
20. Since my relative was diagnosed with incurable illness, I find it more difficult to get along with certain people.	**50.8**	23.7	16.0	7.8	0.8	1.1	1 (1–2)	0.558
21. I wonder what my life would be like if my relative had not been diagnosed with incurable illness.	**32.2**	18.2	20.4	14.4	13.7	1.1	2 (1–4)	0.614
22. I feel more competent since my relative was diagnosed with incurable illness.	**38.2**	31.1	19.6	8.2	1.5	1.5	2 (1–3)	−0.019
23. I get angry when I think about my relative having incurable illness.	**28.9**	23.0	22.6	10.4	14.4	0.8	2 (1–3.5)	0.534
24. Since my relative was diagnosed with incurable illness, I don’t feel interested in keeping up with the day-to-day activities (TV, newspapers, friends).	**45.2**	26.0	19.3	7.4	1.9	0.4	2 (1–3)	0.670
25.I am unable to accept the fact that my relative is diagnosed with incurable illness.	**20.0**	27.0	23.8	17.4	11.5	0.4	3 (2–4)	0.605
26.I am now functioning about as well as before my relative was diagnosed with incurable illness.	11.1	26.0	31.9	21.1	9.6	0.4	3 (2–4)	0.607
27.I am planning for the future.	17.4)	24.1	31.9	13.3	12.6	0.8	3 (2–4)	0.377

a Floor and/or ceiling are marked with bold, defined if more than 20% of the participants used the lowest and/or highest possible scores

b Item-total correlations based on polyserial correlations, adjusted for overlaps

The original AGS total score followed a normal distribution, graphically (P-P and Q-Q plots) and statistically (skewness = 0.15, kurtosis = 2.57, χ^2^(2) = 3.62, *p* = 0.164) (Table 3).

**Table 3:** Scale statistics for the Anticipatory Grief Scale

Factors/Scales	Average score		Score distribution^a^			Score correlation^b^		
	Mdn (q1-q3)	Range	Skewness	Kurtosis	*p*-value	Original AGS	Behavioral reactions	Emotional reactions
Original AGS	73 (65–85)	42–114	0.15	2.57	0.164	1.00		
Behavioral reactions	19 (16–25)	8–35	0.28	2.34	0.003	0.86^***^	1.00	
Emotional reactions	15 (11–19)	5–25	−0.02	2.16	< 0.001	0.72^***^	0.43^***^	1.00

^a^Tested with D’Agostino test

^b^Spearman rank correlations, ^***^ *p* < 0.001

### Homogeneity

The inter-item correlations varied between rho − 0.391 and 0.639, with a mean rho of 0.182. Two problems were identified. First, there were a substantial number of negative correlations (*n* = 65), which ranged between rho − 0.001 and − 0.391 (mean rho = − 0.116). Second, 184 of 286 positive correlations were below rho < 0.3. The positive inter-item correlations varied between rho 0.004 and 0.639 (mean rho = 0.250).

The item-total correlation revealed that 5 items had correlations below 0.3 (item 2, 5, 17, 19 & 22), of which two had negative correlations (item 5 & 22).

### Factor structure

The parallel analysis carried out on the 27 items of the AGS advised that four factors should be retained from the AGS, explaining 52% of the total variance. The GFI of the model was 0.98, indicating an excellent model fit. Eigenvalues of the four factors were all > 1. Despite this, the four-factor model was problematic, because 10 of the 27 items loaded on more than one factor and two items did not load on any factor. Several items also demonstrated weak loadings in all four factors, and 6 had communality values below 0.3 (Table 4). As several of these problems were related to items with unclear directions (i.e. item 2, “I feel close to my relative who has incurable illness”), or were reversely scored, decision was made to evaluate a modified model by omitting these items. In total, 14 items were removed from the AGS.

**Table 4:** Results from the exploratory factor analysis (unweighted least square) of the original Anticipatory Grief Scale (*n* = 270)

Item numbers	Factors and factor loadings^a^				h2^b^
	I	II	III	IV	
1	0.648		0.302		0.523
2				0.685	0.600
3		0.339	0.426		0.343
4			0.619		0.537
5				0.563	0.389
6	0.417		0.346		0.331
7		0.392	0.473		0.443
8			0.414		0.242
9		0.588			0.440
10		0.375	0.677		0.626
11			0.400	0.437	0.391
12	0.445		0.480		0.583
13		0.502		0.545	0.581
14			0.310		0.130
15	0.805				0.666
16			0.552		0.471
17					0.240
18		0.604			0.456
19				0.447	0.221
20		0.731			0.613
21	0.612	0.326			0.521
22					0.241
23	0.776				0.656
24		0.523	0.379		0.498
25	0.707				0.558
26			0.663		0.534
27			0.445		0.230
Explained variance, (%)	26.3	12.1	7.5	6.5	
Cum. Variance, (%)	26.3	38.4	45.9	52.4	

^a^Factor loadings below 0.3 are omitted

^b^Communality values

The parallel analysis of the remaining 13 items in the AGS suggested a two-factor solution with adjusted eigenvalues of 4.68 for the first factor and 1.53 for the second factor, while subsequent factors were < 1. These two factors explained 55% of the total variance. The factor loadings were all strong, ranging between 0.54–0.69 for the first factor, and 0.63–0.82 for the second factor. There were still two items that had double loadings (> 0.3), however the factor loadings were clearly pointing towards one of these two factors. The communalities of the items in the two-factor solution all exceeded 0.3 (Table 5). The GFI of the model was 0.98, indicating excellent model fit. The Kaiser-Meyer-Olkin test (KMO = 0.87) and Bartlett’s test (χ^2^(78) = 1162.5, *p* < 0.001) supported the hypothesis that the data were also suited for a factor analysis for this reduced model of 13 items.

**Table 5:** Results from the exploratory factor analysis (unweighted least square estimation with varimax rotation of the factors) of the modified Anticipatory Grief Scale (*n* = 270)

Item numbers	Factors and factor loadings^a^		h2^b^
	I	II	
Behavioral reactions			
3	0.548		0.343
4	0.537		0.342
7	0.582		0.392
10	0.755		0.587
16	0.578	0.314	0.433
18	0.603		0.377
20	0.691		0.479
24	0.639		0.480
Emotional reactions			
1		0.654	0.488
15		0.816	0.668
21	0.350	0.631	0.520
23		0.727	0.553
25		0.702	0.549
Explained variance (%)	40.0	15.3	
Cum. Variance (%)	40.0	55.3	
Ordinal alpha	0.83	0.84	

^a^Factor loadings below 0.3 are omitted

^b^Communality values

Items 3, 4, 7, 10, 16, 18, 20, and 24 were retained for the first factor, and items 1, 15, 21, 23, and 25 for the second factor. The content of the items was analyzed, and it was found that the first factor was associated with family caregivers’ behavioral reactions to grief (e.g., “I avoid some people since my relative was diagnosed with incurable illness”). Hence this factor was named *Behavioral reactions.* The other factor seemed to be more attributed to the inner and emotional grief reactions (e.g., “I get angry when I think about my relative having incurable illness”). The second factor was named *Emotional reactions.*

### Scale score statistics of behavioral reactions and emotional reactions

In contrast to the original AGS scale, both *Behavioral reactions* and *Emotional reactions* deviated significantly from normal distribution; the scores were not skewed but less peaked than expected for a normal distribution (skewness = 0.28, kurtosis = 2.34, χ^2^(2) = 11.69, *p* = 0.003 vs. skewness = − 0.02, kurtosis = 2.16, χ^2^(2) = 19.17, *p* < 0.001). Graphically, the Q-Q plot showed some deviations in the tails for both scales, while no deviations were detected in the P-P plot. The correlations between *Behavioral reactions* and *Emotional reactions* were moderate (*r*_*s*_ = 0.43) but both correlated strongly with the original AGS score that included all of the items (*r*_*s*_ = 0.86 vs. 0.72, respectively).

### Construct validity

Construct validity was supported for *Behavioral reactions.* The scale was substantially, but not too strongly correlated (< 0.8) with HADS-anxiety (*r*_s_ = 0.70, *p* < 0.001) and HADS-depression *(r*_s_ = 0.65, *p* < 0.001), aligning with the hypothesis that they measure related but different constructs. *Behavioral reactions* also correlated as expected with TRIG I – past behaviors (*r*_s_ = − 0.55, *p* < 0.001) and TRIG II – present feelings (*r*_s_ = − 0.45, *p* < 0.001). For *Emotional reactions,* construct validity was partly supported. The scale correlated weaker than expected with HADS-anxiety (*r*_s_ = 0.36, *p* < 0.001) and HADS-depression (*r*_s_ = 0.27, *p* < 0.001). In contrast, the scale correlated as expected with TRIG I (*r*_s_ = − 0.40, *p* < 0.001) and TRIG II (*r*_s_ = − 0.48, *p* < 0.001).

### Internal consistency

The ordinal alpha for the AGS-scale including all 27 items was 0.91, indicating excellent internal consistency. The traditional Cronbach’s alpha coefficient showed corresponding findings (α = 0.87). The ordinal alpha for the two factors generated from the final factor model was 0.83 and 0.84 for the *Behavioral reactions* and *Emotional reactions,* respectively, indicating that internal consistency was excellent. The corresponding values, measured with the traditional Cronbach’s alpha, were 0.82 and 0.82, respectively (Table 5).

## Discussion

According to our best knowledge, this is the first study that has validated the Anticipatory Grief Scale. The results from the exploratory factor analyses in a sample of 270 family caregivers indicate that, although it has been used in several studies, the original version of the instrument is flawed with regards to its psychometric quality in the context of palliative care. Based on the results of the present study, a modified version of AGS with 13 items over two subscales was suggested which appears to be promising regarding the quality, homogeneity, factor structure and internal consistency of the data. This modified version of the AGS will hereafter be named AGS-13 and include items 3, 4, 7, 10, 16, 18, 20 and 24 to measure *Behavioral reactions*, and item 1, 15, 21, 23 and 25 to measure *Emotional reactions*.

The item score distribution of the original AGS was skewed, with ceiling and floor effects for two-thirds of the items. However, this was not considered to be a serious problem, because the total score of the AGS was normally distributed and all the response options were used and there was a variation in the score distribution. Expressed ceiling and floor effects could influence sensitivity and responsiveness [28]. However, the results indicated no such problems in the original AGS or in the two subscales of the AGS-13, *Behavioral reactions* and *Emotional reactions.* Unlike the original scale, the two subscales both deviated statistically from a normal distribution, but graphically, they were close to a normal distribution.

Few missing values were identified which indicates that the instrument was easy to complete. The item with the highest rate of missing values was number 22 which had a rate of 1.5% missing values, which is decidedly below the level of acceptance of 5%. Item 22 was removed from the AGS-13. In the AGS-13, there were few problems with missing values as none of the items exceeded 0.8% in missing data.

The original AGS demonstrated low homogeneity for several items according to inter-item and item-total correlations. Several items had unclear directions, which could explain the low homogeneity. A further explanation could be that the scale consists of several dimensions, which was also supported by the factor analysis. The results, based on the original AGS, with 27 items, suggested that 4 factors should be retained. However, these factors were inconsistent and difficult to describe conceptually. A possible explanation could be that the AGS includes items with both positive and negative bearing (i.e., reversely scored), something that could lead to greater inconsistencies in the responses, although it has also been speculated that it could reduce acquiescence bias [29]. The poor fit of the original scale to the factor model could also possibly be explained by the fact that the AGS was not originally developed for family caregivers in palliative care. Although the author has stated that the instrument could be used for other diagnoses [30]), it seems as though some items were developed specifically to be used on family caregivers in dementia care (i.e., I feel detached from my relative). Family caregivers with experiences from different care contexts may interpret response options in different ways and their conceptualization of the measured construct could also differ [31]. Unfortunately, there are no other evaluation studies on the AGS and, therefore, it is possible that the original scale also has the same measurement problems in other groups.

In the AGS-13, the 8 items with positive bearing (i.e., reversely scored) and items that were related specifically to family caregivers of patients with dementia were not included. The exclusion of the positive items could be questioned, because they might capture aspects of grief that the remaining, negatively loaded items may not [32]. However, after thorough, consideriations, it was interpreted that the remaining items covered important aspects of the content from the removed items, e.g. feelings towards daily life and mood changes. Earlier research has found that including a few items with reversed directions than the majority of items in the general scale increases the risk of misreading the reversed items, because the person is being asked to shift a mental gear in processing the information [33]. Aligning with the results of this study, reversed items often have lower item-total correlation and they could generally fit less well to factor models [34]. This could invalidate a proposed scale, which could, in fact, be valid and reliable [29]. Items were also removed from the AGS due to weak loadings or unclear directions.

Apart from performing statistical analyses, it is also necessary to review the conceptualization of anticipatory grief over time [31]. According to the constructor, the AGS scale is consistent with existing theoretical and empirical evidence concerning anticipatory grief. However, the scale dates back to 1991 and, of late, the concept has been reconsidered [8]. Anticipatory grief was originally seen as being a part of the total grief work that the family caregiver passed through. In later research, the hypothesis of grief work has been reconsidered and more recent conceptual models of grief describe it as a dual process of both loss and restoration [32]. The two subscales extracted from the AGS-13 were only moderately correlated, which suggests that they would measure two separate, but related constructs.

Construct validity was demonstrated for the AGS-13 subscale *Behavioral reactions* and agreed with our hypothesis that it would be moderately to strongly associated with anxiety, depression and post-death grief. Even though *Emotional reactions* correlated moderately with the TRIG-scales, its correlation with anxiety and depression was weak and construct validity was only partly supported. It could be that *Behavioral reactions* is simply a conceptually stronger subscale. However, it could also be that the concepts measured in *Emotional reactions* (yearning anger, feeling a lack of fairness and inacceptance of the condition) represent a disorder that is essentially different from anxiety and depression.

Reducing the number of items and creating a modified version of the AGS could make the scale more useful in clinical practice as there is a need for instruments that are not only psychometrically sound but also brief and easy to use, especially due to the vulnerability of respondents in palliative care [35]. The two scales of the AGS-13 had somewhat lower internal consistency compared with the original scale. This outcome was expected, as the ordinal alpha, as well as the traditional Cronbach’s alpha, will increase with number of items [36]. Therefore, it can be argued that the internal consistency was even better in the AGS-13, because the two scales included only 8 and 5 item each, compared with the original 27-item version. Further, the alpha level still indicated excellent internal consistency after the instrument was modified.

### Strengths and limitations

This study used exploratory factor analyses because the factor structure of the original AGS was unknown. Hence, the suggested factor model needs to be confirmed in future studies. In total, 14 items were excluded from the AGS, and it is possible that some items were conceptually important in capturing the phenomenon of anticipatory grief. It would seem as though the AGS-13 with its subscales *Behavioral reactions* and *Emotional reactions* measure dimensions of the loss-oriented form of grief rather than restoration-oriented grief. This includes activities and emotions dealing with separation from an attachment figure, and could be compared to the traditional understanding of grief work. The 8 items with positive bearing that were removed from the scale could possibly have captured the more restoration-oriented form of grief [32] and it is also possible that some of the removed items were relevant, not only for dementia, but also in palliative care. The strengths of this validation include that the statistical tests were adapted to ordinal data. Polychoric correlations, a technique to estimate the bivariate correlation between two latent normally distributed continuous variables measured using an ordinal scale [37], are commonly recommended for ordinal data as parametric methods commonly will underestimate the population correlation [38]. The estimation method, ULS with a polychoric correlation matrix, provides accurate factor loadings, and less variable parameter estimates, as well as more precise standard errors compared to other methods ([39] {Li, 2016 #7389). The factor analysis was performed in a sample of 270 family caregivers, which could be considered adequate for the study with a variable ratio of 10:1 [40]. However, a general rule of thumb for exploratory factor analysis is that weak data (low communalities and crossloadings) demands a greater sample [41]. With the AGS-13, items with weak data were removed and the sample size was significantly improved (variable ratio 20:1). However, this validation study needs to be replicated with a larger sample, and there is also a need to validate whether the AGS-13 is invariant across different language versions over time and across different groups, for example with regard to age, sex, and ethnicity and also for family caregivers of patients with different diagnoses in palliative care. It would also be valuable to identify cutoff scores for clinical importance for the AGS-13 which could be used by health care professionals to identify family caregivers in need of support during palliative caregiving.

## Conclusions

In conclusion, it was reasonable to create a modified version of the AGS, as using the original version with its statistical weaknesses could be considered unethical, and it would be inappropriate to overburden family caregivers with unnecessary questions. The AGS-13 version contains fewer items and could be used more easily to capture behavioral and emotional reactions of grief. The AGS-13 might have the potential to be used in future studies of anticipatory grief among family caregivers in palliative care. However, the factor structure needs to be confirmed in further studies.

## Abbreviations

AGS: Anticipatory Grief Scale
GFI: Goodness of Fit Index
KMO: Kaiser-Meyer-Olkin
TRIG: Texas Revised Inventory of Grief
ULS: Unweighted Least Square
